# A New Approach to Medical Records

**Published:** 1990-06

**Authors:** Alison Round

**Affiliations:** 23 Devonshire Place, Exeter EX4 6JA


**LETTER TO THE EDITOR**

**A New Approach to Medical Records**

Sir,

It is, of course, good news that modern technology is being applied to medical record keeping as Professor Pereira Gray and Dr Michael Hall express in their recent article WEMJ. (Mar 1990) 105, 24. I would be interested in how they propose to evaluate the Care Card; presumably patient satisfaction; ease of use by GPs, hospital staff and local pharmacists; utility and efficacy will be amongst the parameters being assessed. I have two areas of concern. Firstly the issue of confidentiality seems to be major, not only in the area of psychological problems but also in the context of maintaining confidentiality within families and friends (who may all have access to a "reader"). Secondly it may be difficult to exclude bias from the evaluation when the investigators so obviously believe that this will be a good way of keeping record (". . . it is possible to envisage all patients in the National Health Service holding their own records in this form"). Is there randomisation between card-holders and non card-holders and are the investigators of patient satisfaction to be blinded as to the kind of records their patients have? In the long-term, this technology, like all other technology, will have to prove its worth in improved patient care.

Alison Round BSc MB MRCP
23 Devonshire Place, Exeter EX4 6JA

